# Intra- and inter-brain coupling and activity dynamics during improvisational music therapy with a person with dementia: an explorative EEG-hyperscanning single case study

**DOI:** 10.3389/fpsyg.2023.1155732

**Published:** 2023-09-29

**Authors:** Clemens Maidhof, Viktor Müller, Olivier Lartillot, Kat Agres, Jodie Bloska, Rie Asano, Helen Odell-Miller, Jörg Fachner

**Affiliations:** ^1^Cambridge Institute for Music Therapy Research, Anglia Ruskin University, Cambridge, United Kingdom; ^2^Josef Ressel Centre for Personalized Music Therapy, University of Applied Sciences IMC Krems, Krems an der Donau, Austria; ^3^Center for Lifespan Psychology, Max Planck Institute for Human Development, Berlin, Germany; ^4^RITMO Centre for Interdisciplinary Studies in Rhythm, Time and Motion, University of Oslo, Oslo, Norway; ^5^Yong Siew Toh Conservatory of Music, National University of Singapore, Singapore, Singapore; ^6^Centre for Music and Health, National University of Singapore, Singapore, Singapore; ^7^Institute of Musicology, University of Cologne, Cologne, Germany; ^8^Advanced Comprehensive Research Organization, Teikyo University, Tokyo, Japan

**Keywords:** music therapy, improvisation, hyperscanning, EEG, music information retrieval

## Abstract

**Objective:**

Real-life research into the underlying neural dynamics of improvisational music therapy, used with various clinical populations, is largely lacking. This single case study explored within-session differences in musical features and in within- and between-brain coupling between a Person with Dementia (PwD) and a music therapist during a music therapy session.

**Methods:**

Dual-EEG from a music therapist and a PwD (male, 31 years) was recorded. Note density, pulse clarity and synchronicity were extracted from audio-visual data. Three music therapists identified moments of interest and no interest (MOI/MONI) in two drum improvisations. The Integrative Coupling Index, reflecting time-lagged neural synchronization, and musical features were compared between the MOI and MONI.

**Results:**

Between-brain coupling of 2 Hz activity was increased during the MOI, showing anteriority of the therapist’s neural activity. Within-brain coupling for the PwD was stronger from frontal and central areas during the MOI, but within-brain coupling for the therapist was stronger during MONI. Differences in musical features indicated that both acted musically more similar to one another during the MOI.

**Conclusion:**

Within-session differences in neural synchronization and musical features highlight the dynamic nature of music therapy.

**Significance:**

The findings contribute to a better understanding of social and affective processes in the brain and (interactive) musical behaviors during specific moments in a real-life music therapy session. This may provide insights into the role of such moments for relational-therapeutic processes.

## Introduction

An important part of music therapy practice is joint musical improvisation, often used with various clinical populations to help alleviate symptoms and induce therapeutic change and psychosocial effects (for an overview of improvisation methods and techniques, see Bruscia, 1987; Wigram, 2004). For example, improvisational music therapy has been reported to be helpful in stress reduction and the management of behavioral and psychological symptoms of dementia (such as aggression, agitation, and apathy; Hsu et al., 2015; Abraha et al., 2017; Sittler et al., 2021), to improve anxiety and depression (Erkkilä et al., 2011, 2021; Fachner et al., 2013), and to be beneficial for children with autistic spectrum disorder (Mayer-Benarous et al., 2021). In contrast to other musical activities, anyone can engage in some form of musical improvisation, for example, no musical training is required by participants, and clinical improvisations may have no aesthetically pre-defined constraints or goals. Rather, a focus is often placed on the interpersonal relationship between the client and therapist, in which the spontaneous, moment-by-moment co-creation of music can form the basis for non-verbal communication. Besides music’s important ability to fulfil social needs, which has been stressed in the music psychology literature (Koelsch, 2013), the underlying mechanisms by which musical improvisation can lead to physical as well as mental health and communication benefits have been grouped into four areas: (a) unconscious expression, (b) creative expression, (c) creative social interaction, and (d) emotional expression (MacDonald and Wilson, 2014). However, research into the underlying neural dynamics of improvisational music therapy is largely lacking, although understanding socio-affective and neurocognitive mechanisms by which therapeutic change occurs could be a crucial step toward a better knowledge about the action mechanisms of music therapy interventions (Fachner, 2014). In addition, there is an increased interest in social neuroscience in musical interaction, often seen as an ideal model to study social interaction (e.g., Babiloni et al., 2011; for reviews, see e.g., Babiloni and Astolfi, 2014; D’Ausilio et al., 2015; Acquadro et al., 2016; Müller et al., 2021; Müller, 2022).

Drumming appears to be an ancient form of non-verbal communication, with drums found in China dating back ca. 5500-2350 BC (Liu, 2005), and manual drumming being even observable in the African great apes (Fitch, 2006). Improvisation on percussion instruments is very common in music therapy, probably because no specific fine motor skills are required (Nordoff and Robbins, 1977), its ability to communicate emotional content (Rojiani et al., 2018), and the reduced complexity in the absence of melodic and harmonic features. A previous study that focussed on drumming with clinical populations reported improvements in depression, anxiety, and social resilience, as well as in immune system responses (Fancourt et al., 2016). Other studies, e.g., in trauma- and addiction-related work, highlighted aspects such as an increased sense of togetherness, connectedness, and sense of self-control after group drumming (Winkelman, 2003; Bensimon et al., 2008; Faulkner, 2017). In non-clinical populations, there is additionally a considerable interest in interactional or interpersonal synchrony and synchronized movements (including drumming and tapping) and their underlying neural mechanisms (e.g., Kokal et al., 2011; for reviews, see Trost et al., 2017; Hoehl et al., 2020; Schirmer et al., 2021). These studies suggest that experimentally manipulated synchronous actions can have various social benefits and effects, such as prosocial behavior, perceived social bonding, social cognition, and positive affect (for a review, see Mogan et al., 2017). Therefore, we focus in this explorative EEG case study on the participant’s free drum improvisations during a music therapy session, whilst the music therapist uses percussion to musically support.

Findings from studies using hyperscanning, i.e., the method to record brain activity from multiple interactants at the same time, have led to the view that playing music together requires strong interbrain synchronization and specific hyperbrain network activity supporting interpersonal action coordination (Lindenberger et al., 2009; Sänger et al., 2011, 2012, 2013; Müller et al., 2013, 2018; Müller and Lindenberger, 2019, 2022). Two recent EEG-hyperscanning studies in naturalistic music therapy settings investigated neural coupling between child-parent and child-therapist dyads (Samadani et al., 2021; Kang et al., 2022). In one study, it was reported that neural synchronization between a non-participating, covertly observing parent and their child was increased during music therapy, compared to a baseline period, and that interbrain synchrony increased over the time period of a therapy session (Samadani et al., 2021). In another study (Kang et al., 2022), significant interbrain synchronization between a child and music therapist was found, which however did not differ between the music therapy sessions and storytelling sessions (although child-parent synchronization was increased during music compared to storytelling). These studies provided important information about neural coupling across therapy sessions but did not analyze the musical content and did not differentiate in more detail between segments during the therapy sessions. This could provide important information, as previous work has shown that spontaneously jointly created improvisations in music therapy can be reflective of physical, cognitive, emotional, and social functioning of clients (Luck et al., 2006). For example, Foubert et al. (2017) found that clients with borderline personality disorder play less synchronized music with the therapist, probably related to their insecure attachment system. In contrast, another hyperscanning study in receptive music therapy focused on certain moments during a session that were rated as being therapeutically more interesting and involved, e.g., a pivotal moment that initiated a transition in the client (Fachner et al., 2019). Focusing on certain moments followed the practice that often particular parts of session(s) are selected to describe a narrative of therapeutic change processes in music therapy (Bruscia, 1991; Hibben, 1999; Aldridge, 2005). These target sequences in a time series of clinically relevant events that are recognized and selected based on personal and/or professional preferences and interests have been termed “Moments of Interest” (Fachner, 2017), and there is growing research interest into specific moments during joint improvisation and their role for therapeutic and relational processes (Hadar and Amir, 2021; Smetana et al., 2022). The results of that hyperscanning study (Fachner et al., 2019) showed that the frontal alpha asymmetry (FAA), a marker of emotional processes (Smith et al., 2017; for reviews, see e.g., Allen et al., 2018; Reznik and Allen, 2018), differed between these “Moments of Interest” and moments that were therapeutically less interesting. In addition, peaks in the time course of the FAA during particular moments seemed to be aligned between client and therapist, which might indicate shared emotional processing (Fachner et al., 2019). This indicates the importance of investigating specific events and within-session differences, based on the highly dynamic social-musical interactive contents of therapy sessions.

However, studies in active, improvisational music therapy that investigate in more detail changes in interbrain coupling within sessions are still lacking. Therefore, the aims of this single case study were to (a) explore interbrain synchronization between a music therapist and a Person with Dementia (PwD) during drum improvisations that were of different therapeutic importance, (b) explore quantitative descriptors of the improvisations, and (c) explore the FAA as a neural marker of emotional processes during different parts of a therapy session. In this way, it is possible to gain insights into social and affective processes in the brain and (interactive) musical behaviors during significant/meaningful moments in a music therapy session. The goal of this explorative single case study was thus not to generate generalizable findings, but to provide an in-depth exploration of a real-life single session that includes the details of participants’ natural musical behaviors and situational dynamics of a therapy session, playing a crucial role for music therapy research (Fachner, 2014). This study thus potentially can generate hypotheses for future research and can serve as a starting point for further single case studies, small-n studies and larger-n studies that explore these hypotheses further.

## Materials and methods

### Participants

The client was a 31 year old male with impairments in memory, speech, and motor skills due to a rare form of familial early-onset Alzheimer’s disease, probably caused by mutations in the *PSEN1* gene, encoding presenilin-1 (PS1) (Kelleher and Shen, 2017; Sun et al., 2017). He participated in a BBC TV documentary about music and dementia entitled “Our Dementia Choir” that followed several people living with dementia while they took part in a dementia choir (Our Dementia Choir, 2019). Part of the documentary also featured research into music and dementia, and the Cambridge Institute for Music Therapy Research (CIMTR) at Anglia Ruskin University (ARU) was approached by the producers for information about music therapy and brain research. Following telephone assessment, and ethical procedures, the aforementioned client from Our Dementia Choir consented, and was very keen to take part in the research, accompanied by his wife. On the day of data recording, the client and his wife and a small TV crew came to the music therapy center at ARU. During the music therapy session, a camera operator and a microphone operator remained in the background of the music therapy room. The session took place in a room that was set up typically for a music therapy session (in addition to EEG equipment), with tuned and untuned percussion instruments, a piano, guitar and transverse flute. The session was observed by his wife, two researchers, the host of the documentary (Vicky McClure), and a camera operator from an observation room. The therapist was a 28-year-old female with experience in working with people with dementia, including clients with Alzheimer’s disease (J.B.).

Participants, neither of whom lacked capacity to consent to taking part in research, gave informed written consent prior to the study, which was approved by the local ethics committee of Anglia Ruskin University and conducted in accordance with the Declaration of Helsinki.

### Description of the session

In preparation for the one-off session, the music therapist called with the participant’s wife to determine symptoms and musical preferences of the PwD. She then identified the following broad aims: (a) encourage verbal and non-verbal self-expression, (b) promote social interaction with and without the use of words, (c) promote the use of unimpaired cognitive functions (such as memory, attention, etc.) and provide cognitive stimulation, and (d) enable reminiscence and strengthen self-identity.

The session lasted ca. 25 min and was preceded and followed by eyes-closed EEG resting-state recordings (each ca. 5 min) of both client and therapist. Table 1 shows the duration and details of the different parts of the session, which consisted mainly of improvisations on various instruments and the performing of two personally meaningful songs (as determined during a pre-assessment over the phone with the PwD’s wife). The song “Use somebody” by Kings of Leon (2008) had been chosen as a favorite song, which the client knew very well and was related to meeting his wife for the first time (it was described as “their song” by his wife). The song “Stand by me” (King, 1961) was chosen because it was part of a planned performance with the dementia choir.

**TABLE 1:** Overview of different parts of the music therapy session.

Part	Duration	Comment	Instruments
(Drum) Improvisation	5:57 min	Improvisation with a theme about the journey coming to the Music Therapy Centre and the situation there	PwD plays floor tom with two felt beaters; MT plays conga
“Use Somebody” (Kings of Leon)	4:27 min	Personally meaningful song	PwD sings, taps with his feet and hands, plays conga during the second half (after encouragement by the therapist); therapist sings and plays guitar
Improvisation	2:59 min		PwD plays conga; therapist plays transverse flute
(Drum) Improvisation	2:55 min		PwD plays conga; therapist plays tabla (Bayan)
“Stand by me”	4:01 min	Personally meaningful song	PwD plays conga; therapist sings and plays guitar

The two improvisations during which both participants played drums consisted each of three parts, with short breaks in between where the musical interaction came to an end.

### Selection of segments

Three music therapists, including the involved therapist, acted as raters. They watched the video recording independently and were asked to identify the three most interesting moments during the session (“Moment of Interest,” MOI), i.e., a segment that they thought is interesting or important in the current therapeutic context, of any length. Additionally, they were instructed to identify one to three “moments of no/less interest” (“MONI”). All raters had clinical experience in working with PwD (range: 3–39 years).

**FIGURE 1:**
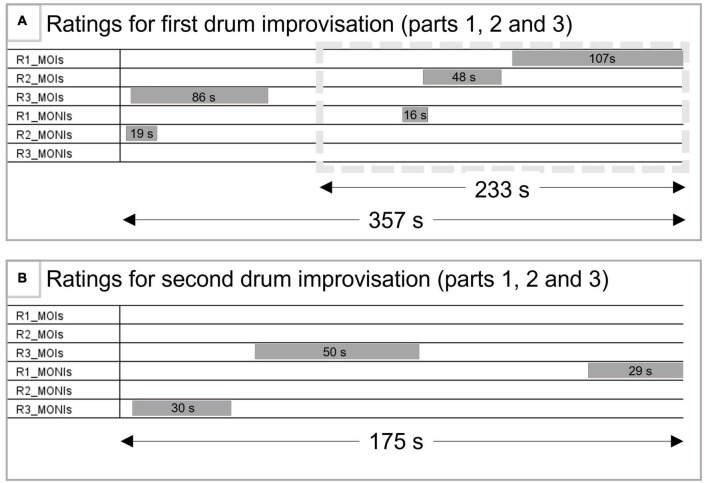
Ratings of the session. **(A)** Depicts the selected moments during the first drum improvisation. The gray box (dotted line) shows the part of this drum improvisation used for analysis of within- and between-brain coupling. **(B)** Depicts the selected moments during the second drum improvisation, also used for within- and between brain coupling analyses. R1-3 = rater 1 (therapist of the session) to rater 3; MOIs, moment of interest; MONIs, moments of no interest.

As can be seen in Figure 1, all three raters identified moments of interest during the first drum improvisation. In addition, two out of three raters identified shorter moments of no interest during that improvisation, partly overlapping with MOIs from other raters. In contrast, the second drum improvisation seemed to be less interesting to therapists: Two out of three raters, including the involved therapist, selected segments of this improvisation as being of no/less interest (and none of them selected a Moment of Interest during this part). However, the third rater selected one segment as an interesting moment. In sum, the results of this particular rating procedure (asking for “Moments of Interest” without further specifications) suggested that no single moment during the session was easily identified as being interesting to all raters. However, 67.5% of the duration of the first drum improvisation was rated as interesting, while only 9.8% was rated as not or less interesting. In contrast, only 28.6% of the duration of the second drum improvisation was rated as interesting (only by one therapist), while 33.7% was rated as not interesting. In addition, the first drum improvisation included more therapeutically interesting parts compared to the last drum improvisation. Based on these individual rating results, showing a relative difference in how the two improvisations were rated, we therefore classified the first drum improvisation as a Moment of Interest (MOI) and the second drum improvisation as a Moment of No Interest (MONI). For the remainder of this paper, we thus refer to the first drum improvisation as a MOI and to the second drum improvisation as a MONI. Because the calculation of the brain coupling measures required comparable amounts of EEG data in terms of length, we only used parts 2 and 3 (3:53 min) of the first drum improvisation and all three parts of the second drum improvisation (2:55 min). Even though this did not result in equal durations of MOI and MONI, by doing so we avoided introducing artificial boundaries in naturally unfolding improvisations, as well as including the very first musical contact of the whole session.

### Data recording and analysis

#### Audio, video, and musical data

First, an audio file from the music therapy session was converted to MIDI using Logic Pro software. More specifically, the session’s audio file was used as input to Logic Pro, and the musical events (created by both the PwD and therapist) were manually segregated into separate MIDI tracks for the PwD and therapist. To ensure the accuracy of the transcription, the MIDI tracks were then imported into ELAN software (Wittenburg et al., 2006) and, based on video and audio data, corrected if necessary. Note that determining the exact onsets of drum sounds was not always possible, due to the overlapping sounds generated by the simultaneously played instruments. However, with the aid of video, audio waveform and spectrogram, onsets could be detected within a window of approximately ± 1–2 frames, corresponding to ± 40–80 ms.

Drum onsets were further analyzed in Matlab (Mathworks, Inc.) with the Music Therapy Toolbox (Luck et al., 2006, 2008), itself based on the MIDI toolbox (Eerola and Toiviainen, 2004). Several features were extracted from the data, including note density, pulse clarity, pulse synchronicity, and imitation. Whereas note density and pulse clarity relate to one single player, synchronicity and imitation describe the interaction between two players. The feature analysis was carried out using a sliding window of fixed length (6 s). The temporal location of the window’s end point was changed with steps of 1 s, measured from the first note onset of each stimulus. Each of the four features is described below.

*Note density* was computed for each window as the number of notes divided by the length of the window. This feature therefore provides a measure of the moment-by-moment rhythmic density of the improvised music and is calculated for each player separately.

The *pulse clarity* feature measures the degree to which the client and therapist produce periodic musical events (as measured during each 6 s analysis window), i.e., the momentary salience of the beat or pulse. To illustrate the difference between note density and pulse clarity, one can imagine a musical sequence containing many notes (resulting in high note density) but without any rhythmical regularity, e.g., with random note onsets (resulting in low pulse clarity). Conversely, a musical sequence with few notes but with perfectly equal durations and regular note onsets would result in low note density but high pulse clarity. A sequence with high note density and high pulse clarity would contain a group of very short/fast notes at each beat and sub-beat level. To calculate the value of this variable, a temporal function was first constructed by summing Gaussian kernels centered at the onset points of each note. The height of each Gaussian kernel was constant; the SD was set to 30 ms (see Toiviainen and Snyder, 2003). Subsequently, the obtained function was subjected to autocorrelation using temporal lags between 250 ms and 1,500 ms, corresponding to commonly presented estimates for the lower and upper bounds of perceived pulse sensation. To model the dependence of perceived pulse salience on beat period, the values of the autocorrelation function were weighted with a resonance curve having the maximal value at the period of 500 ms (see also Van Noorden and Moelants, 1999; Toiviainen, 2001). The maximal value of the obtained weighted autocorrelation function was taken to represent the degree of instantaneous pulse clarity. Pulse clarity is defined as the maximum autocorrelation value along the autocorrelation function. Like all the other musical parameters, the autocorrelation function and pulse clarity were computed for each successive position of the sliding window. The analysis of a complete improvisation results in a two-dimensional diagram called a pulsation diagram (Luck et al., 2006), where each successive column corresponds to a successive frame. The evolution of client’s and therapist’s pulse clarity is thus obtained by collecting the maximal values of each successive column in the respective pulsation diagram.

*Pulse synchronicity*, related to the individual pulse clarity values, measures the degree to which the client and therapist played synchronously to a common beat/pulse. For example, if one player shows low note density while the other player shows high note density, but both have high pulse clarity, synchronicity will be high if the tempo of each player is the same. If the tempo is different (without a clear ratio between the tempi), then synchronicity will be low. If any of the two players has low pulse clarity, then synchronicity will be low as well. To assess the common pulsation developed in synchrony by both players, another diagram called a synchronized pulsation diagram was produced by multiplying each individual player’s values at respective points of their related pulsation diagrams. Similarly to individual pulse clarity, the evolution of synchronicity is given by the maximal pulsation values in the synchronized pulsation diagram.

A related feature that can be derived from the note density and pulse clarity measures is *imitation*, which measures the degree to which one player reproduces (at a small time-lag) the relative temporal evolution of either note density or pulse clarity of the other player’s musical improvisation. If the time-lag decreases to zero, the two players become aligned with respect to this specific dimension. Indeed, the degree of communication between the therapist and the client is another important dimension of musical expression that is of particular interest in music therapy. One form of engagement and communication is when players imitate one another or jointly elaborate the same gestures (De Backer, 2004; Wigram, 2004) to create a musical dialogue, drawing on the innate musicality of human communication (Malloch and Trevarthen, 2009), which may be easier for a PwD to engage in than verbal conversation. The musical dialogue may therefore be assessed by observing the degree of local similarity between the temporal evolutions of both improvisations, along the different features presented in the previous section. These local similarities are represented graphically in an imitation diagram (Luck et al., 2006; see Figure 3 below). In this type of diagram, the horizontal axis corresponds to the temporal evolution of the improvisation. Lines in the imitation diagram indicate local imitation. The brightness of the lines is associated with strength of imitation: dark-blue corresponds to slight and coarse similarities, while bright yellow corresponds to distinct and temporally-close imitations. When the line is vertically centered, the imitation between both players is synchronous. When the horizontal line is in the upper half of the diagram, the client imitates the therapist after a certain delay, displayed by the vertical axis, in seconds. Similarly, when the line is in the lower half of the diagram, the therapist imitates the client. Finally, the length of the line indicates the duration of the imitation.

Note density, pulse clarity and pulse synchronicity were each statistically analyzed using non-parametric repeated measurement ANOVAs due to the non-normal distribution of the data, using R (version 4.1.2.; R Core Team, 2021) and the nparLD package (Noguchi et al., 2012). ANOVA-type statistics (ATS) were computed with between-subject factor Role (PwD/therapist) and within-subject factor Moment (interesting/non-interesting). Because of the unequal lengths of the improvisations during the interesting and non-interesting moment, we created new time series of equal length by taking the average of three consecutive data points during the MOI, and the average of two consecutive data points during the MONI. Visual inspection showed that the global trends were preserved in these filtered versions of the time series.

#### EEG

The EEG was recorded with 500 Hz from 32 Ag/AgCl active electrodes (ActiCap, Brain Products GmbH, Germany) for each participant, placed according to the extended 10–20 system (FP1, FP2, F7, F8, F3, F4, Fz, FT9, FT10, FC5, FC6, FC1, FC2, T7, T8, C3, C4, TP9, TP10, CP5, CP6, CP1, CP2, Pz, P7, P8, P4, P3, Oz, O1, O2), referenced to Cz of participant 1. To enable free movements of the participants, we used the MOVE interface in combination with an ActiCHamp amplifier (Brain Products GmbH, Gilching, Germany). This system enables wireless EEG data transmission by connecting electrode cables to transmitters that were attached to the shoulders/back of the participants. As there was no physical reference electrode for participant 2, the first pre-processing step involved re-referencing the EEG signals of participant 2 to electrode Cz of participant 2. The ground electrodes were placed on Fpz on each participant and were recorded by using a ground distributor connected to the amplifier. Simultaneous video and audio were recorded with a Sony HVR Z1E camera, connected via Firewire to a PC running the BrainVision Video Recorder software (Brain Products GmbH, Germany) that synchronized video and EEG signals. Data were offline first analyzed with the BrainVision Analyzer software (version 2.1.2; Brain Products GmbH). Data were filtered with a 4th order Butterworth IIR filter (1–40 Hz; 50 Hz notch) and eye movements were corrected using Independent Component Analysis. Other artifacts such as head and body movements were rejected by visual inspection.

#### Frontal alpha asymmetry

For the analysis of the frontal alpha asymmetry (FAA), the influence of non-frontal alpha sources (potentially unrelated to motivation/emotion such as occipital alpha sources) on alpha activity recorded over frontal leads (Allen and Reznik, 2015; Allen et al., 2018) was reduced by a current source density (CSD) transformation. Data were then divided into 1 s epochs with 0.5 s overlap and artifactual epochs were automatically detected and manually checked (based on scalp-recorded voltage fluctuations). Automatic rejection criteria were: maximal 50 μV voltage step, maximal allowed difference of 200 μV in 200 ms intervals, absolute threshold of ± 75 μV, and lowest activity of 0.5 μV in 100 ms intervals. A Fast Fourier Transformation (FFT) was performed (max. resolution 1 Hz) on all artifact-free epochs, which were Hanning windowed (10%). Power values for all Fourier coefficients were exported and further analyzed in Matlab (version 9.2.0, Mathworks, Inc.). Alpha power for each segment was calculated as the band (8–13 Hz) value sum and the alpha asymmetry scores were computed by taking the differences between the natural-log transformed power values of frontal electrodes F4 and F3. Mean FAA values were calculated based on all epochs.

#### Coupling analyses

Procedures for analyzing inter- and intra-brain coupling followed previous studies (Müller et al., 2013, 2018; Sänger et al., 2013; Müller and Lindenberger, 2019). Data were re-referenced to an average of left and right mastoid electrodes, resampled at 250 Hz using a spline interpolation method, and then divided into artifact-free 10 s epochs for MOI (parts 2 and 3 of the first drum improvisation) and MONI (second drum improvisation) parts, respectively. Due to artifacts, five out of 23 segments for the MOI and three out of 17 segments for the MONI were discarded.

Subsequent time-frequency and coherence analyses were performed with a LabView-based tool developed by Viktor Müller (cf. Müller and Lindenberger, 2011; Müller et al., 2013; LabView, National Instruments, Austin, Texas, USA). EEG time series were transformed into a complex time-frequency signal using a complex-valued Morlet wavelet with a time resolution of 4 ms and frequency resolution of 0.125 Hz. From this, the instantaneous phase differences between all 4,096 possible electrode pairs within and between the brains for 12 frequencies were computed (2, 4, 6, 8, 10, 12, 14, 16, 18, 20, 24, 28 Hz).

The Integrative Coupling Index (ICI), an asymmetric index of in-phase synchronization, was derived in several steps. First, we separately counted the data points at which phase angle differences were in the range between -π/4 and 0 and between 0 and + π/4, respectively (phase angle differences beyond those ranges were regarded as points of non-synchronization). Before counting, successive points that fell in either of the ranges but for a time interval shorter than a period of the corresponding oscillation at the given frequency were discarded or explained to non-synchronization points, thereby eliminating instances of accidental synchronization. This resulted in the Negative and Positive Coupling Indices (NCI and PCI, respectively), representing the relative number of phase-locked points in the negative and positive range, respectively. The Absolute Coupling Index (ACI) indicates the relative number of data points in both, negative and positive ranges. Finally, the ICI was calculated by the formula (Müller and Lindenberger, 2011):

ICI = ((PCI + ACI)/(2 × ACI)) × *sqrt*(PCI)

The ICI is equal to 1 when all points are phase-locked with a positive phase angle difference and approaches 0 when all points are phase-locked with a negative phase angle difference or are beyond the range of -π/4 to + π/4. Thus, the ICI is an asymmetric index and indicates the anteriority of one time series phase over another. The ICI was calculated for each electrode pair (within and between-brains) and for each trial.

#### EEG data reduction and statistical analyses

In a first explorative step, we calculated coupling (ICI) between all electrodes within- and between-brains and determined coupling strength or out-strength as the sum of weights of all outgoing connections for each electrode also separately for within- and between-brain connections. For MOI and MONI, we constructed hyper-brain networks, consisting of the therapist’s and client’s brains. These were then presented in the form of the connectivity matrix and connectivity maps as well as in the form of corresponding topological distributions of strengths also within and between brains. For statistical analyses, we determined coupling strengths by summing ICI values over electrode pairs for three Regions of Interest (ROI), covering the whole head (frontal: Fp1, Fp2, F7, F3, Fz, F4, and F8; central: FC1, FC2, C3, Cz, C4, CP1, and CP2; parietal: P7, P3, Pz, P4, P8, O1, and O2). That is, the ICI values of 49 electrode pairs were summed for each possible connection between two ROIs of the two participants (between-brain coupling) and for each possible connection between two ROIs within each participant (within-brain coupling). This was done for each frequency bin, and then averaged across trials, separately for the MOI and MONI. Results suggested differences only for the 2 and 4 Hz frequency bins, and only those were further analyzed. Note that this result is in line with previous research on guitarists playing in duet (Lindenberger et al., 2009; Sänger et al., 2012; Müller et al., 2013; Müller and Lindenberger, 2019, 2022).

Statistical analyses (in SPSS, version 26, IBM, USA) consisted of repeated measures ANOVAs. For the coupling measures, ANOVAs (14 cases, each representing coupling strengths for a 10 s segment) with factors Moment (MOI, MONI), Direction (from therapist to PwD, from PwD to therapist), and Site (frontal, central, parietal) were calculated. For within-brain coupling, the factor Direction was replaced by the factor Role (therapist, PwD). For the FAA, mean FAA values were initially calculated based on all epochs. However, due to the different number of epochs for each part of the session, statistical analyses were conducted on a random subset of 321 epochs (number of segments of the shortest part), for each session part. Statistically significant (alpha level: *p* = 0.05) effects were followed up by *post hoc* ANOVAs or pairwise comparisons. Reported *p*-values were corrected using the Greenhouse-Geisser method when the assumption of sphericity was violated (as tested with Mauchly’s test of sphericity) and estimates of epsilon < 1. Pairwise comparisons were made using a Bonferroni correction.

For further explorative correlation analyses (Kendall’s tau) between coupling, musical interaction parameters, and affective state, we averaged musical synchronicity as well as the FAA in the best-matching 10 s time windows as the EEG coupling measures were derived from. Because the scalp maps of interbrain coupling, especially for 2 Hz, suggested nominally stronger effects over frontal electrodes (see Section “Results”), we focussed on the frontal ROI of both participants.

## Results

### Comments of raters

After watching the video recording, the therapist commented overall on the session that: “*Being a musician, D can see the decline in his drumming since the onset of his dementia. He was very focused on playing ‘correctly,’ although this could at times be difficult for him; he was very aware of when he was struggling to stay on the beat. This seemed to be related to initiating the arm movement to begin playing the drum – once he found a steady beat he was able to play continuously to a steady beat and incorporate different rhythms. This was evident when he repeated what the therapist played back, stopping in between his playing, as his playing could be slightly delayed. Once playing continuously though, this was not as observable.”*

#### First drum improvisation

Furthermore, the therapist commented that during the first segment she selected as MOI (during the first drum improvisation, part 3; see Figures 1, 3), the improvisation felt disjointed (for a few seconds) after a brief period of back-and-forth mirroring, and that it was difficult for the PwD to follow. However, introducing a steady beat by the therapist allowed for joint playing which seemed more “relaxed” and “together” for the following ca. 100 s.

The second rater labeled her selected MOI during the first drum improvisation (part 2) as a meaningful dialogue between therapist and client. She described repeating rhythms and a movement toward a shared musical climax and “cadence together with a changing emotional quality toward the middle to the end of the moment,” and that there was a “playful exchange between them.” According to this rater, this was remarkable as client and therapist had not met before and it was their first musical interaction, “so it is especially meaningful to see their eye contact and change of rhythms in a dialogue leading up to the cadence.”

In addition, the involved therapist also selected a 16 s sequence as not being interesting. This was a sequence toward the end of part 2 where both were “*drumming together but client-therapist ‘lose’ each other and go out of sync, which brings the improvisation to an end.”*

#### Second drum improvisation

The therapist commented on the third part of this improvisation (selected as a Moment of No Interest) that it feels a bit “forced” and that the client seemed to have lost interest, that he is not very engaged in this. The third rater noted that the first part of the improvisation “*might not be particularly important as it repeated an activity that had been done earlier during the session*,” but rated the second part as interesting, due to a change in the therapist’s playing (accentuating fewer beats) that seemed to have helped the client to maintain a regular rhythm.

### Musical analyses

**FIGURE 2:**
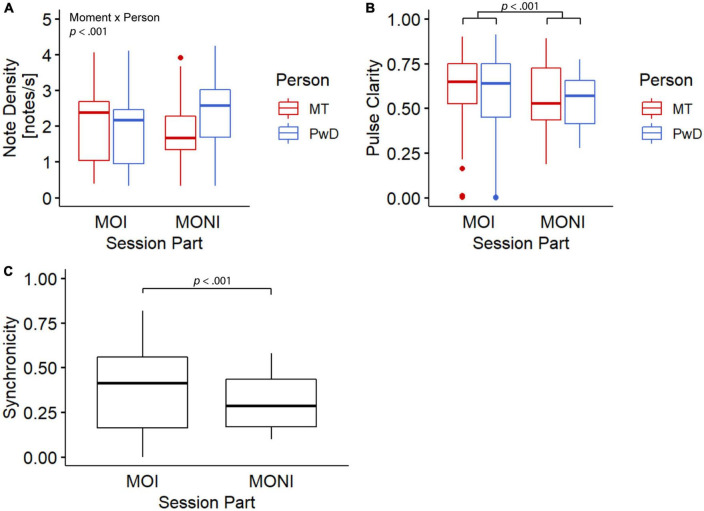
Boxplots for note density **(A)**, pulse clarity **(B)**, and note synchronicity **(C)**. MT, music therapist; PwD, person with dementia; MOI, moment of interest; MONI, moment of no interest.

**FIGURE 3:**
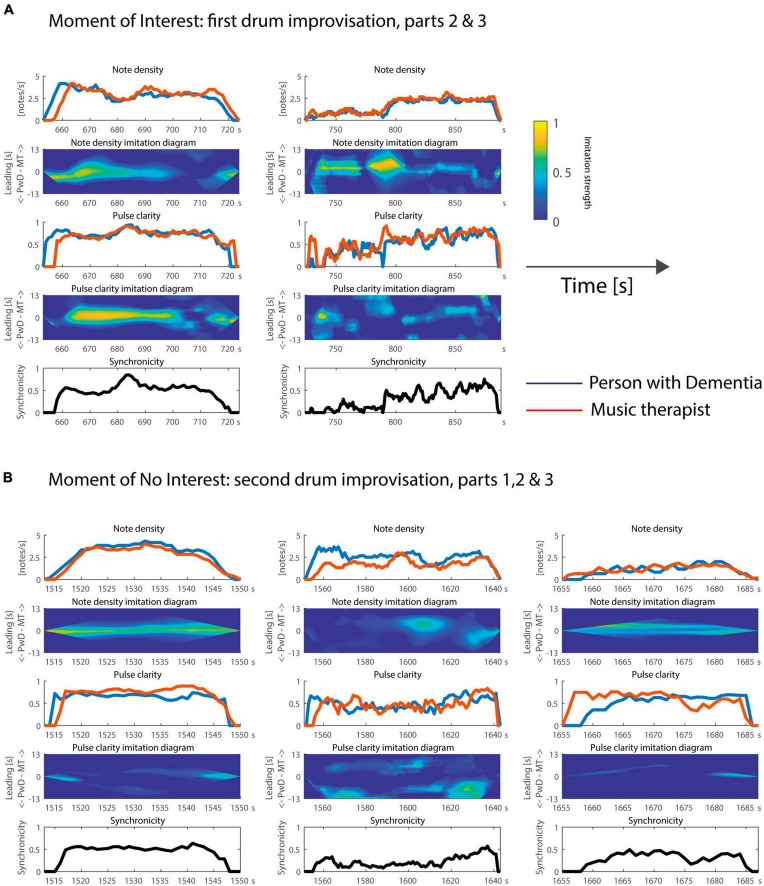
Time courses of note density, pulse clarity, and note synchronicity during the **(A)** Moment of Interest and **(B)** Moment of No Interest. In addition, the note density and pulse clarity imitation diagrams indicate who is following or leading during the improvisations. Time is plotted on the x axis as elapsed time since the start of the video recording in seconds.

Figures 2, 3 show the means and time courses of note density, pulse clarity, and note synchronicity, respectively. Statistical analyses showed that the note density of the music therapist differed from the note density of the Person with Dementia, and this difference was more pronounced during the Moment of No Interest (see Figure 2A). An ANOVA-type statistics (ATS) with within-subject factor Moment (interesting, non-interesting) and between-subject factor Person (PwD, MT) showed a significant interaction between Moment and Person [ATS(1) = 15.72, *p* < 0.001] and a marginally significant effect of Person [ATS(1) = 3.41, *p* = 0.06]. On average, the pulse clarity (see Figure 2B) was higher during the MOI compared to the MONI, but did not differ between PwD and MT. An ANOVA-type statistics showed only a main effect of Moment [ATS(1) = 17.72, *p* < 0.001], and no interaction between Person and Moment [ATS(1) = 0.58, *p* = 0.44]. Similarly, synchronicity (Figure 2C) was significantly higher during the MOI compared to the MONI [significant main effect of Moment: ATS(1) = 11.94, *p* < 0.001].

Based on the temporal evolution of these features and the imitation diagrams (depicted in Figure 3), several additional observations can be made.

Looking first at the MOI, one can see from the note density feature for part 2 that the client leads the musical interaction at the beginning, and then the two players are more aligned (both the PwD and therapist’s note density evolves in the same way at the same time). At the end of part 2, the PwD leads again with respect to note density: he decides to stop playing at a particular point in time, and the therapist then ends her playing shortly thereafter. Based on the video, it seems the client was attempting to repeat a rhythm introduced by the MT, but he stops playing after struggling to maintain the rhythm and tempo due to motor impairments. Both client and therapist have to laugh after the part comes to an end. For pulse clarity, one can see a similar trend, except that the therapist leads with a very small time lag during the middle part. This seems to relate to the peak around 680–690 s, which is initiated by the therapist and followed by the client. Finally, in terms of synchronicity, the pair seem to synchronize quite well (again with a peak around 680–690 s) to a common pulse. Compared to part 2, the note density is smaller during part 3. The two players imitate each other with respect to the temporal evolution of note density throughout the whole part. During a large section, the therapist leads, introducing musical changes, such as the significant increase of note density around 790 s, which are quickly followed by the PwD. At this point, after a section of mirroring/following of rhythmic patterns introduced by the therapist, the therapist starts to play a steady beat, and shortly after, the PwD joins in and they play synchronously for several seconds. The leader role later changes, with the PwD initiating the change in note density for the ending. In terms of pulse clarity, the improvisation was in general a bit more chaotic than the previous part, but the yellow and green clouds in the imitation diagram indicate particular moments of slight imitations: some quick changes are rather synchronized in the beginning, the therapist leads around 790 s, and then they imitate each other throughout the second half of this part, first in a synchronized manner, and then with the therapist as a leader. Finally, we can see from the synchronicity diagram that their individual pulsations before 790 s do not follow a common pulse, but generally do afterward. This might be related to the fact that the PwD seems to be slightly delayed in repeating what the therapist plays; it appears to be challenging for him to repeat newly introduced patterns.

Now we turn to the MONI. For the first part, the client is slightly ahead in note density, then both players are aligned, and finally at the end the therapist is slightly ahead. For pulse clarity, the client is clearly ahead at the beginning, then the players become somewhat independent until they become aligned at the end. Based on the video, the independent development of pulse clarity between 1,535–1,540 s seems to be related to the client’s problems with bimanual coordination, something that can be observed in other parts as well. In general, note density, pulse clarity, and synchronicity tend to be high between 1,520 to 1,540 s, but this is a very brief excerpt. For the second part, the PwD initiates a new rhythmic pattern, creating a higher note density than the therapist. There is no clear imitation until the middle of this part, where a decrease in note density by the therapist is imitated by the PwD. The final decrease in note density seems to be initiated, with a slight lead, by the PwD. For pulse clarity, the yellow cloud at the beginning of the imitation diagram seems to indicate that the client was leading the improvisation, and the same can be observed at the very end of this part. The low synchronicity here indicates that their individual pulsations did not share a common pulse, except at the end of that part. For the third part, concerning note density, both players alternate in the form of a call and response, initially started by the therapist, and ending in a rather synchronized way, but slightly led by the PwD, according to our model. They do not show much imitation with respect to the temporal evolution of pulse clarity, except for the fact that the start and end of this part is led by the therapist. Although playing alternately, the two musicians do share a common pulse, as shown by the moderate synchronicity.

### Inter- and intra- brain synchronization

**FIGURE 4:**
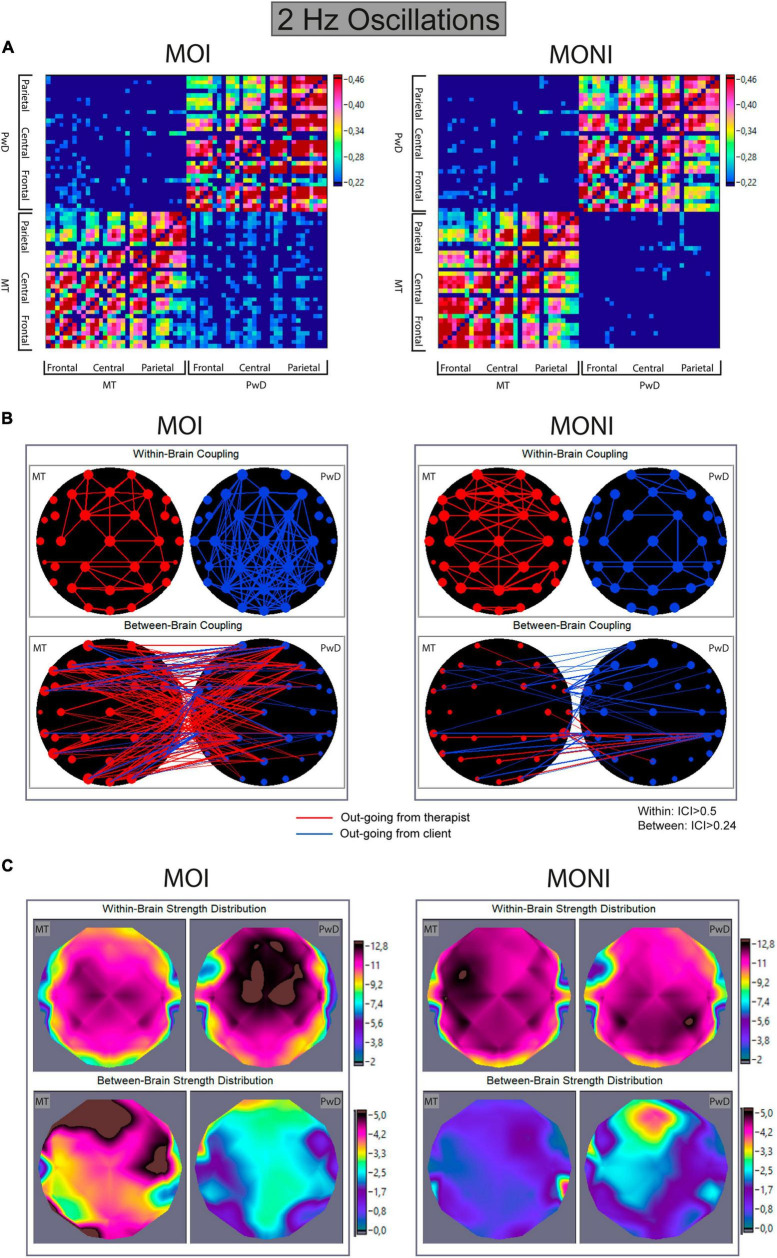
Within- and between-brain coupling for 2 Hz oscillations during the Moment of Interest and Moment of No Interest. **(A)** Connectivity matrices with all significant intra- and interbrain connections for the Integrated Coupling Index (ICI). The network includes 32 nodes (electrodes) of the music therapist’s brain and 32 nodes of the client’s brain. The connection strength is indicated by colors ranging from dark blue (threshold connectivity values) to dark red (high values). Within-brain coupling of the therapist is captured in the lower left, and within-brain coupling of the client is captured on the upper right quadrant. Between-brain coupling is captured in the lower right (out-going connections from therapist to client) and upper left quadrant (out-going connections from client to therapist). **(B)** Connectivity maps. The upper panel represents the connectivity within the brain of the therapist (MT) and the client (PwD), and the lower panel represents the connectivity between brains. The strength of the nodes (sum of all out-going connections) is coded by circle size, and the strength of edges is coded by the width of the line. Red lines represent out-going connections from the therapist, and blue lines out-going connections from the client. **(C)** Topological distribution of the strengths. The upper two maps represent the topological distribution of the out-strengths within the brain of the therapist (MT) and client (PwD), and the lower ones display the topological distribution of the out-strengths going from the therapist to the client (left) and vice versa (right).

**FIGURE 5:**
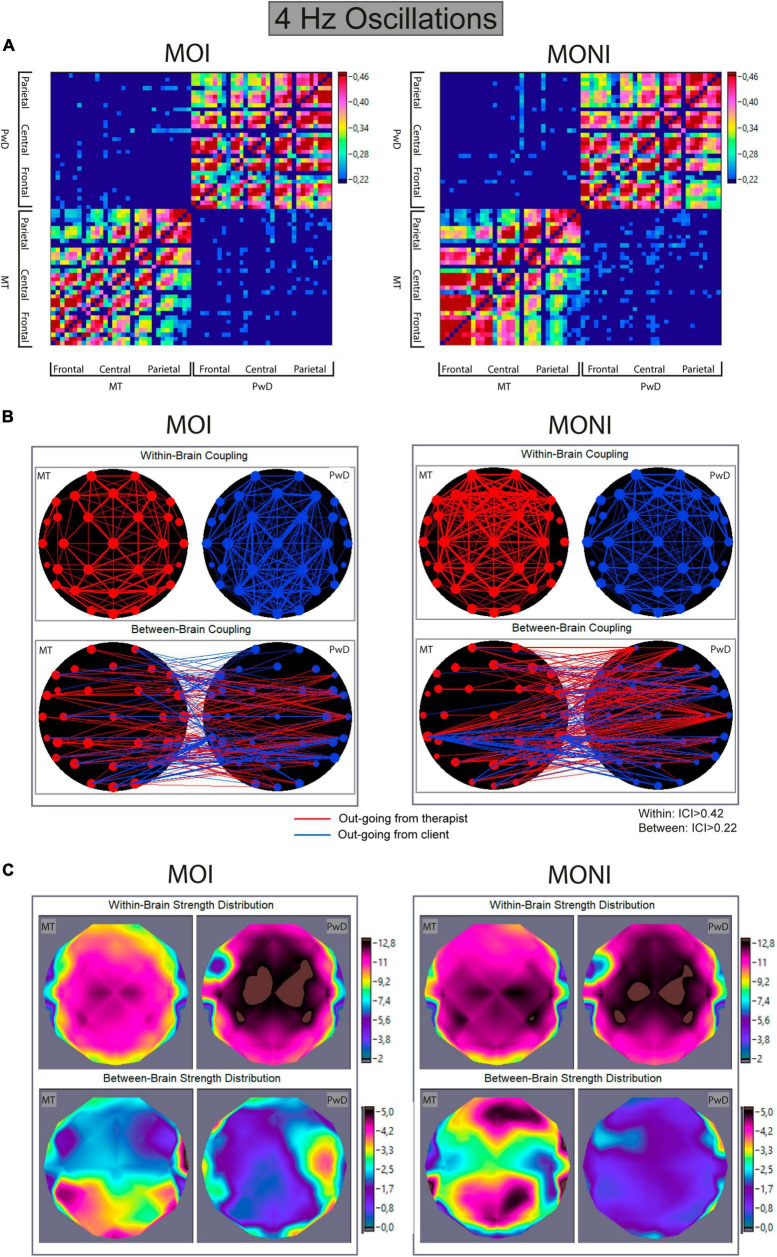
Within- and between-brain coupling for 4 Hz oscillations during the Moment of Interest and Moment of No Interest. **(A)** Connectivity matrices with all significant intra- and interbrain connections for the Integrated Coupling Index (ICI). The network includes 32 nodes (electrodes) of the music therapist’s brain and 32 nodes of the client’s brain. The connection strength is indicated by colors ranging from dark blue (threshold connectivity values) to dark red (high values). Within-brain coupling of the therapist is captured in the lower left, and within-brain coupling of the client is captured on the upper right quadrant. Between-brain coupling is captured in the lower right (out-going connections from therapist to client) and upper left quadrant (out-going connections from client to therapist). **(B)** Connectivity maps. The upper panel represents the connectivity within the brain of the therapist (MT) and the client (PwD), and the lower panel represents the connectivity between brains. The strength of the nodes (sum of all out-going connections) is coded by circle size, and the strength of edges is coded by the width of the line. Red lines represent out-going connections from the therapist, and blue lines out-going connections from the client. **(C)** Topological distribution of the strengths. The upper two maps represent the topological distribution of the out-strengths within the brain of the therapist (MT) and client (PwD), and the lower ones display the topological distribution of the out-strengths going from the therapist to the client (left) and vice versa (right).

Figures 4, 5 show the connectivity matrices, connectivity maps, and distributions of strengths during the MOI and MONI for 2 Hz and 4 Hz oscillations, respectively. Each connectivity matrix shows the within- and between-brains synchronization patterns across all possible electrode pairs for therapist and client representing hyper-brain connectivity network, where only the strongest connections are shown (see Figures 4A, 5A). In the connectivity maps (Figures 4B, 5B), the individual connections between electrodes are coded by the thickness of the link and the out-strengths are coded by the size of the electrodes. The topological distribution of the out-strengths as represented in Figures 4C, 5C is indicated by color. It can be seen that coupling strengths and their topological distributions in therapist and client appear to differ between the Moment of Interest and No Interest.

To further quantify these observations, we summarized coupling strengths for frontal, central, and parietal ROIs and conducted ANOVAs with factors Moment (interesting, non-interesting), Direction (from therapist to PwD, from PwD to therapist), and Site (frontal, central, parietal).

#### Directed out-strengths in between-brain networks

2 Hz. An ANOVA showed a marginally significant main effect of Moment [*F*(1,13) = 3.8, *p* = 0.07, η^2^p = 0.23] and a significant interaction between Moment and Direction [*F*(1,13) = 5.16, *p* = 0.04, η^2^p = 0.28]. A follow-up ANOVA (collapsed across Site) showed a marginally significant effect of Direction only during the MOI [*F*(1,13) = 4.08, *p* = 0.064, η^2^p = 0.24], indicating that the between-brain out-strengths from the therapist to the PwD were increased during the MOI compared to the MONI (see Figures 4B, C).

4 Hz. An ANOVA showed a significant main effect of Direction [*F*(1,13) = 7.9, *p* = 0.015, η^2^p = 0.38], but no further main effects or interactions. This indicated that the out-strengths from the therapist to the client, compared to from the client to the therapist, were increased, but that this effect did not differ between the different session parts (see Figure 5).

Results of additional correlation analyses revealed a marginally significant positive correlation between musical synchronicity and frontal out-strengths from client to therapist for 2 Hz (*τb* = 0.294, *p* = 0.09) only during the MOI, indicating that 2 Hz coupling from client to therapist was stronger when musical synchronicity was higher. There were no associations between FAA and interbrain coupling results.

#### Directed out-strengths in within-brain networks

2 Hz. An ANOVA showed main effects of Role [*F*(1,13) = 6.81, *p* = 0.022, η^2^p = 0.34] and Site [*F*(2,26) = 44.503, *p* < 0.0001, η^2^p = 0.77], and interactions between Role and Moment [*F*(1,13) = 12.966, *p* = 0.003, η^2^p = 0.5] and between Role, Moment, and Site [*F*(1.34,17.36) = 6.693, *p* = 0.013, η^2^p = 0.34]. Follow-up analyses separately for each participant showed main effects of Moment [*F*(1,13) = 6.79, *p* = 0.022, η^2^p = 0.34] and Site [*F*(2,26) = 13.6, *p* < 0.001, η^2^p = 0.51] for the therapist. For the client, an ANOVA showed a marginally significant main effect of Moment [*F*(1,13) = 4.4, *p* = 0.056, η^2^p = 0.25] and of Site [*F*(2,26) = 17.19, *p* < 0.0001, η^2^p = 0.57], and an interaction between Moment and Site [*F*(1.36,17.71) = 5.71, *p* = 0.02, η^2^p = 0.31]. This indicated that within-brain coupling in the therapist was higher during the MONI, compared to the MOI, and that coupling was stronger over central/frontal areas (see Figures 4B, C). For the client, within-brain coupling from frontal and central areas appeared to be stronger during the MOI, whereas it was stronger during the MONI from parietal areas.

4 Hz. An ANOVA showed main effects of Role [*F*(1,13) = 76.56, *p* < 0.0001, η2p = 0.86] and Site [*F*(1.36,17.69) = 61.2, *p* < 0.001, η^2^p = 0.83], and an interaction between Role and Moment [*F*(1,13) = 22.40, *p* < 0.0001, η^2^p = 0.63]. A follow-up ANOVA separately for each participant (collapsed across Site) showed a main effect of Moment only for the therapist [*F*(1,13) = 8.43, *p* = 0.012, η2p = 0.39]. This suggests that compared to the MOI, within-brain out-strengths were increased during the MONI for the therapist, but not for the client (see Figure 5).

### Frontal alpha asymmetry

**FIGURE 6:**
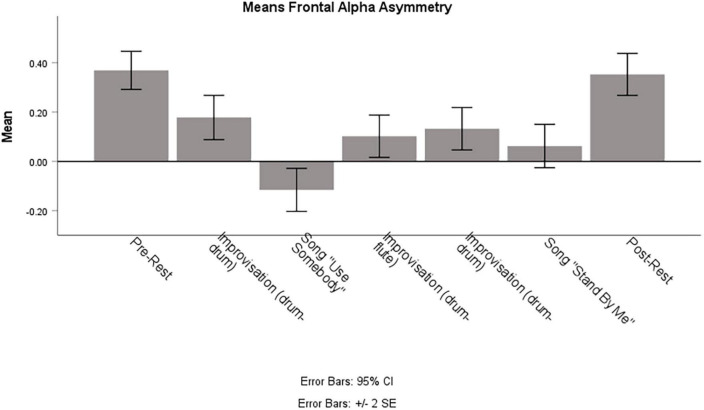
Means of Frontal Alpha Asymmetry of the Person with Dementia during different parts of the session. Note that means are calculated on a random subset of 321 epochs, which corresponds to the number of segments of the shortest session part.

Figure 6 shows the averaged FAA values of the PwD for each part of the therapy session. A repeated measures ANOVA showed that the FAA differed between the different parts of the session [*F*(6,1920) = 15.51, *p* < 0.0001, η^2^p = 0.046]. Bonferroni-corrected pairwise comparisons showed that the FAA did not differ between the MOI and MONI, and it did not differ between the pre- and the post-resting state recordings. However, the FAA during both resting-state recordings was significantly higher when compared to each session segment (except that there was no significant difference between post-rest and the first drum improvisation). In addition, only the FAA during the song “Use Somebody” showed a negative mean, which was significantly lower compared to all other session parts, except that the nominal difference to the mean during the song “Stand By Me” did not reach statistical significance.

## Discussion

This single case EEG-hyperscanning study explored inter- and intra-brain synchronization during an active, improvisational music therapy session with a Person with Dementia. In addition, we quantified musical features of free improvisations during the therapy session. We focused on two drum improvisations that appeared to differ in terms of therapeutic interest, as determined by ratings and comments from the involved therapist and two other music therapists, who identified “Moments of Interest” (MOI) and “Moments of No/Less Interest” (MONI). For example, the involved music therapist selected a Moment of Interest during the first drum improvisation when a steady beat was introduced by the therapist which allowed for joint playing that seemed more “relaxed and together.” An example of a selected MONI was in the second drum improvisation toward the end of the session where the improvisation “felt a bit forced and the client seemed to have lost interest.” Of importance in this case study is also the fact that the PwD is a former drummer and he was thus “very focused on playing correct” and was “very aware of when he was struggling to stay on the beat” (therapist notes – see above).

These observations from a therapeutic perspective appear to be consistent with the computational analysis of musical features. We found that musical synchronicity, i.e., the degree to which the client and therapist played synchronously to a common beat/pulse, was overall higher during the MOI compared to the MONI. Consistent with this, the pulse clarity of each interactant was higher during the MOI but did not differ between the players. In addition, the difference between client and therapist in note density, i.e., a measure of the moment-by-moment rhythmic density, was more pronounced during the MONI. This indicates that both acted musically more similar to one another (more musically connected) during the MOI. Inspection of the temporal evolution of these features allowed identification of changes in musical behaviors, such as imitation, changes in leader-follower roles, and peaks in musical synchronicity.

Importantly, we also found differences between the MOI and MONI regarding neural coupling between the PwD and the therapist. This was investigated with the ICI (Müller and Lindenberger, 2011), which indicates the anteriority of one time series phase over another and thus reflects time-lagged neural synchronization. Results showed that between-brain coupling of 2 Hz oscillatory activity going from the therapist to the client was increased during the MOI compared to the MONI. Simultaneously, within-brain coupling for the PwD was stronger from frontal and central areas during the MOI, but within-brain coupling for the therapist was stronger during the MONI. In contrast, for 4 Hz oscillatory activity, between-brain coupling from the therapist was increased (compared to from the client) but did not differ between the session parts. Similarly to the 2 Hz results, within-brain coupling for the therapist during the MOI was lower, but no differences between the session parts for the client were found.

Whereas no significant differences in topology for between-brain coupling were found, within-brain coupling at 2 Hz revealed stronger coupling from frontal and central areas during MOI and MONI for the therapist, and different network topographies for MOI (frontal/central areas) and MONI (parietal) for the PwD. As noted by Lindenberger et al. (2009) and also Sänger et al. (2012), the prominent role of fronto-central brain regions is consistent with the assumption that the representation of one’s owns and the other person’s actions in real time and their partial integration into a joint, inter- personally shared forward model may help to initiate, sustain and maintain interpersonal action coordination.

The finding of neural coupling in low frequency oscillations is consistent with previous studies investigating interpersonal action coordination during music improvisation (Müller et al., 2013; Müller and Lindenberger, 2022). Given that synchronization in the present study occurred mainly in the 2 Hz frequency range, which is roughly the tempo of the improvisations, a direct link to the produced music seems plausible, resembling ‘delta-wave entrainment to external periodic acoustic stimulation’ (Will and Makeig, 2011) as an induced rhythm of the brain(s). However, the current study did not explore mere listening-related entrainment but a jointly created drum rhythm in a tempo range of about 2 Hz. Thus, interbrain coupling in 2 Hz could be a form of neural entrainment to the rhythmic structure of the improvisations (Nozaradan, 2014; Nozaradan et al., 2015; Zoefel et al., 2018), and differences in coupling strengths could be explained in terms of differences in musical content of the improvisations and related differences in sensorimotor coordination (perhaps also indicated by the positive correlation between the client’s out-strengths and musical synchronicity, but only during the MOI). In an EEG hyperscanning study with pianist duets, a delta/theta inter-brain synchronization (IBS) increase was found when partners had received opposite tempo instructions and were slightly less behaviorally synchronized. However, the authors attributed this IBS increase not to timekeeping *per se*, but to the “compensatory increase of attention to the partner and mutual adaptive behavior upon detection of the subtle temporal mismatches between self-and other-produced sounds” (Gugnowska et al., 2022). On the other hand, a recent study reported that neural coupling in the delta band (but not in the theta band) during a non-interactive situation was increased after a dyadic musical interaction (Khalil et al., 2022). It has been argued that this effect, potentially linked to the musical beat, might reflect a mechanism by which social interaction and a sense of affiliation could be maintained after an interruption. In addition, it has been shown that affective and social gestures increase delta interbrain connectivity, compared to informative gestures (Balconi and Fronda, 2020). Thus, one might speculate that an alternative explanation for the present findings could be that different musical coordination patterns combined with different (higher-level) interaction qualities and dyadic experiences (e.g., shared empathetic responses, social connectedness, quality of the relationship and therapeutic alliance) might have led to the observed differences in neural coupling between the two improvisations.

Some of these interaction qualities have also been mentioned by the raters, who commented (unprompted) not only on the music itself but also on aspects of non-musical communication (e.g., eye contact) and level of engagement of the client in the interaction. Given that more individual moments of interest during the first improvisation were selected by the raters (leading to the classification of the two improvisations into a MOI and MONI), this interpretation would be consistent with research that focuses on the emergence of meaningful moments within a client-therapist relationship during joint improvisation (e.g., Fachner, 2014; Coomans, 2018; Fachner et al., 2021; Hadar and Amir, 2021; Smetana et al., 2022). It would also be in line with a recent study into the therapeutic use of music that reported higher neural synchronization during music compared to storytelling in lower frequency bands over frontal areas in a non-speaking youth with cerebral palsy and her parent that was observing the session (Kang et al., 2022). The authors argue that their pattern of results might be related to socio-emotional responses in the parent, while the therapist might have performed primarily on a cognitive level.

Importantly, the above-described higher-level interaction qualities and dyadic experiences occurred during a clinical improvisation, in which a therapist aims to support the client in their playing (especially at the start of a session). Therefore, the therapist’s attention is governed by therapeutic intentions:

*…there is a difference between music-making that is geared simply to playing music with someone else…. As therapists, we clinically observe and analyze the client’s areas of musical strengths and limitations, of avoidances and resistances, and the music we improvise is shaped to work with this, to help the client move into dealing with the areas of difficulty, as well as enabling a free expression and sharing through the musical interaction* (Brown and Pavlicevic, 1996, 404).

Thus, the intention to focus attention on the client’s playing may allow to speculate that the increased directed coupling from the therapist during the MOI as compared to during the MONI, as indicated by the anteriority of the therapist’s neural activity, may reflect this therapeutic attitude “of maintaining an objective and clinical viewpoint in order to preserve for the patient the creative potential of the therapeutic space” (ibid). An indicator for this might be seen in part 2 of the first drum improvisation (MOI; see section musical analysis), in which the patient leads the beginning of the improvisation (and the therapist is attending to his play) compared to the middle, where both are more aligned (in terms of note density) and the therapist supports the playing, being aware of him “struggling to stay on the beat” and she leads in terms of pulse clarity with a small time-lag, which then evolves into a peak in pulse clarity and synchronicity (around 685 s). Also, in part 3 we can see this supporting intervention (offering a steady beat) from 790 s onward, where the therapist takes the client’s left arm movement limitations into account by keeping the pulse. Before that a call and response type intervention revealed his limitation to play a steady beat alternatingly with both arms.

The MONI seems to represent not only a continuation of the problems related to PwD’s bimanual coordination and how the therapist relates to it, but here the focus has changed from an anamnestic attitude and attentive openness to the participant’s abilities during the MOI (being aware that the client is a drummer), toward an understanding of the coordination problems which resulted in “…client-therapist ‘lose’ each other and go out of sync…” (from therapist’s notes). During the MOI the therapist offered a supportive rhythmical framework with a ‘steady beat’ increasing musical synchronicity (which may be reflected in the increased 2 Hz coupling), but this therapeutic intervention was not in focus anymore during the MONI. The MONI represents a therapeutic mode of dealing with the result of the anamnestic process (“struggling to stay on the beat”), which might explain the apparent finding of no differences in direction (from therapist, from client) of 2 Hz between-brain coupling during the MONI. However, it can be assumed that the therapist maintained her therapeutic attention toward the client during both the MOI and MONI, which may be expressed in the main effect of direction as seen in the 4 Hz between-brain coupling during MOI and MONI (see Figure 5B). In short, one might speculate that the therapeutic interaction during drumming might have been related to the 2 Hz coupling, while maintaining therapeutic relation and attention might have been related to the 4 Hz coupling. However, more research is clearly needed to investigate these speculations before any conclusions regarding the relationships between ideographic clinical observations and within-and between-brain coupling and activity dynamics can be drawn. Lastly, a potential effect of decreased sustained attention and/or fatigue, especially in this client population, cannot be excluded and should be taken into account in future studies.

Because of the importance of emotion in music and music therapy, we also investigated a neural marker of affective processes, i.e., the frontal alpha asymmetry (Smith et al., 2017; for reviews, see e.g., Allen et al., 2018; Reznik and Allen, 2018). In contrast to the findings regarding neural coupling, the FAA did not differ between MOI and MONI. This could indicate comparable affective processes in both improvisations, and that the observed differences in inter- and intra-brain coupling in this study do not reflect changes in socio-affective processes. However, it might also be the case that the used averaging approach concealed potential differences and important temporal dynamics of affective processes (e.g., shorter periods of alignment), especially given that musical improvisation is a highly dynamic interaction and consisted in our case of several instances of role change as well as break downs and re-starting the improvisation. Nevertheless, the FAA only during the song “Use Somebody” showed a negative mean amplitude and was significantly lower compared to almost all other session parts. This observation might be related to the autobiographical memories of a significant life event associated with this song and the current difficulties faced by the PwD and his family (the wife in the observation booth was crying during this song).

### Further implications for future research

The results of this single case study cannot be generalized to a wider population nor across therapy sessions as they are primarily specific to this music therapy session. Therefore, results need to be replicated with a larger sample or with series of case studies. Interestingly, in music neuroscience, there has recently been a plea for case studies “to gain a deeper, more nuanced understanding of creative musical processes” (Barrett and Limb, 2019, 88), and a recent fMRI single case study has provided important insights into brain activation and connectivity during music improvisation (Barrett et al., 2020). There it has been argued that “averaging artists in group experimental studies may eliminate key neural variability that contributes to an artist’s unique creative abilities” (Barrett et al., 2020, 2) and that case study research alongside group studies is a possible way to develop neural models of creativity (Barrett et al., 2020). An analogous argument can be made for case studies in neuroscientific research into music therapy, as creative and therapeutic processes will be highly individualistic and specific for the given therapeutical and situated context.

In contrast to our previous single case study into a receptive form of music therapy in which clients verbalized their experiences and emotions (Fachner et al., 2019), we did not find strong agreements of all raters on segments of the session. Future studies should include client-selected moments and other qualitative data (de Witte et al., 2021), and the feasibility of this approach has already been demonstrated with another client population (Tucek et al., 2022). It might also be a fruitful approach to combine the open concept of “interesting moments” and neuroscientific data in general with criteria in relation to therapeutic goals, client population-specific criteria, or therapeutic/change factors such as therapeutic alliance and empathy, and theory-driven mechanisms of change. Embedded in longitudinal designs that also apply outcome measures, this could help to derive at a neuroscientific-based change process research, especially if real-world case studies that contextualize rich data of naturally occurring interactions are accompanied with naturalistic, more controlled experiments, including with non-clinical populations (Schilbach, 2016; Matusz et al., 2019; Crum, 2021).

Although this study combined EEG data with video and musical-behavior analyses, future studies should look at the temporal evolution of musical features and interactional behaviors. For example, combining temporally-resolved EEG data analysis methods (both in terms of neural coupling as well as neuro-affective markers) with analyses of the temporal evolution of interactional behaviors and musical features (following a micro-analytic approach; see Wosch and Wigram, 2007) could significantly improve our understanding of the moment-by-moment fluctuations during social interactions, including further insights into the exact relationship between neural dynamics (also in frequency bands beyond 2 and 4 Hz) and musical parameters–the correlation reported here between client’s out-strengths and musical synchronicity might be a fruitful starting point. Furthermore, future studies could investigate simultaneously different types of synchrony measures, e.g., measures based on amplitude alignment (e.g., Zamm et al., 2018) or measures that can capture non-linear relationships, such as Mutual Information, and their relation to dynamic interpersonal behavior. In addition, using electronic instruments and/or high-quality audio recordings would allow the analysis of loudness and duration also of non-percussive instruments, as well as acoustic features such as sensory dissonance, which can reveal important insights into improvisations in music therapy (Luck et al., 2006; Kurkjian et al., 2021).

In sum, this is the first study to demonstrate the feasibility of assessing directed inter- and intra-brain coupling in a real-life setting of an improvisational music therapy session with a Person with Dementia, combined with musical and qualitative data. Even though the exact functional significance of neural coupling remains uncertain, the finding of increased inter-brain synchronization and increased musical synchronicity during therapeutically interesting moments in a single music therapy session represent an important first step for further research into social neuroscience of music therapy. This line of research may help to better understand therapeutic mechanisms and processes, in turn enhancing outcomes for clients. It may also enhance understanding of brain functions during musical interactions with others such as for people living with dementia and their carers, and for music therapists working in this field. In the future, it is also conceivable that such multimodal data might provide information about ongoing intra- and inter-individual processes, potentially allowing for more effective session reviews or enabling therapists to adaptively respond even during sessions.

## Data availability statement

The raw audio data supporting the conclusions of this article will be made available by the authors, without undue reservation. There is no consent from the participants for their raw EEG and video data to be shared. Requests to access the datasets can be directed to the corresponding author.

## Ethics statement

The studies involving humans were approved by the Departmental Research Ethics Panel (Department for Music and Performing Arts) at Anglia Ruskin University. The studies were conducted in accordance with the local legislation and institutional requirements. The participants provided their written informed consent to participate in this study. Written informed consent was obtained from the individual(s) for the publication of any potentially identifiable images or data included in this article.

## Author contributions

CM and JF conceptualized the study and collected the data. CM, VM, OL, KA, HO-M, JB, and JF analyzed the data. CM, OL, KA, VM, and JF drafted the manuscript. All authors reviewed and revised the draft critically and contributed to the article and approved the submitted version.

## References

[B1] Abraha I. Rimland J. M. Trotta F. M. Dell’Aquila G. Cruz-Jentoft A. Petrovic M. (2017). Systematic review of systematic reviews of non-pharmacological interventions to treat behavioural disturbances in older patients with dementia. The SENATOR-OnTop series. *BMJ Open* 7:e012759. 10.1136/bmjopen-2016-012759 28302633 PMC5372076

[B2] Acquadro M. A. S. Congedo M. De Riddeer D. (2016). Music performance as an experimental approach to hyperscanning studies. *Front. Hum. Neurosci.* 10:242. 10.3389/fnhum.2016.00242 27252641 PMC4879135

[B3] Aldridge D. (ed.) (2005). *Case study designs in music therapy.* London: Jessica Kingsley Publishers.

[B4] Allen J. J. B. Reznik S. J. (2015). Frontal EEG asymmetry as a promising marker of depression vulnerability: Summary and methodological considerations. *Curr. Opin. Psychol.* 4 93–97. 10.1016/j.copsyc.2014.12.017 26462291 PMC4599354

[B5] Allen J. J. B. Keune P. M. Schönenberg M. Nusslock R. (2018). Frontal EEG alpha asymmetry and emotion: From neural underpinnings and methodological considerations to psychopathology and social cognition. *Psychophysiology* 55:e13028. 10.1111/psyp.13028 29243266

[B6] Babiloni C. Vecchio F. Infarinato F. Buffo P. Marzano N. Spada D. (2011). Simultaneous recording of electroencephalographic data in musicians playing in ensemble. *Cortex* 47 1082–1090. 10.1016/j.cortex.2011.05.006 21664610

[B7] Babiloni F. Astolfi L. (2014). Social neuroscience and hyperscanning techniques: Past, present and future. *Neurosci. Biobehav. Rev.* 44 76–93. 10.1016/j.neubiorev.2012.07.006 22917915 PMC3522775

[B8] Balconi M. Fronda G. (2020). The use of hyperscanning to investigate the role of social, affective, and informative gestures in non-verbal communication. Electrophysiological (EEG) and inter-brain connectivity evidence. *Brain Sci.* 10:29. 10.3390/brainsci10010029 31948108 PMC7017113

[B9] Barrett K. C. Limb C. J. (2019). Unveiling artistic minds: Case studies of creativity. *Curr. Opin. Behav. Sci.* 27 84–89. 10.1016/j.cobeha.2018.09.005

[B10] Barrett K. C. Barrett F. Jiradejvong P. Rankin S. K. Landau A. Limb C. J. (2020). Classical creativity: A functional magnetic resonance imaging (fMRI) investigation of pianist and improviser Gabriela Montero. *NeuroImage* 209:116496. 10.1016/j.neuroimage.2019.116496 31899286

[B11] Bensimon M. Amir D. Wolf Y. (2008). Drumming through trauma: Music therapy with post-traumatic soldiers. *Arts Psychother.* 35 34–48. 10.1016/j.aip.2007.09.002

[B12] Brown S. Pavlicevic M. (1996). Clinical improvisation in creative music therapy: Musical aesthetic and the interpersonal dimension. *Arts Psychother.* 23 397–405. 10.1016/S0197-4556(96)00033-0

[B13] Bruscia K. (1987). *Improvisational models of music therapy.* Springfield, IL: Charles C Thomas.

[B14] Bruscia K. (ed.) (1991). *Case studies in music therapy.* Gilsum, NH: Barcelona Publishers.

[B15] Coomans A. (2018). Moments in Music Therapy – A review of different concepts and connotations in music therapy. *Musikther. Umsch.* 39 337–353. 10.13109/muum.2018.39.4.337

[B16] Crum J. (2021). Understanding mental health and cognitive restructuring with ecological neuroscience. *Front. Psychiatry* 12:697095. 10.3389/fpsyt.2021.697095 34220594 PMC8249924

[B17] D’Ausilio A. Novembre G. Fadiga L. Keller P. E. (2015). What can music tell us about social interaction? *Trends Cogn. Sci.* 19 111–114. 10.1016/j.tics.2015.01.005 25641075

[B18] De Backer J. (2004). *Music and Psychosis: The transition from sensorial play to musical form by psychotic patients in a music therapeutic process.* Dissertation. Aalborg: Aalborg University.

[B19] de Witte M. Orkibi H. Zarate R. Karkou V. Sajnani N. Malhotra B. (2021). From therapeutic factors to mechanisms of change in the creative arts therapies: A scoping review. *Front. Psychol.* 12:27. 10.3389/fpsyg.2021.678397 34366998 PMC8336579

[B20] Eerola T. Toiviainen P. (2004). *MIDI toolbox: MATLAB tools for music research.* Jyväskylä: University of Jyväskylä.

[B21] Erkkilä J. Brabant O. Hartmann M. Mavrolampados A. Ala-Ruona E. Snape N. (2021). Music therapy for depression enhanced with listening homework and slow paced breathing: A randomised controlled trial. *Front. Psychol.* 12:613821. 10.3389/fpsyg.2021.613821 PMC7920974 33664693

[B22] Erkkilä J. Punkanen M. Fachner J. Ala-Ruona E. Pöntiö I. Tervaniemi M. (2011). Individual music therapy for depression: Randomised controlled trial. *Br. J. Psychiatry* 199 132–139. 10.1192/bjp.bp.110.085431 21474494

[B23] Fachner J. (2014). Communicating change – meaningful moments, situated cognition and music therapy: A response to North (2014). *Psychol. Music* 42 791–799. 10.1177/0305735614547665

[B24] Fachner J. (2017). “Music, moments, and healing process: Music therapy,” in *The routledge companion to music cognition*, eds Ashley R. Timmers R. (Abingdon: Routledge), 89–99. Available online at: https://www.routledge.com/The-Routledge-Companion-to-Music-Cognition/Ashley-Timmers/p/book/9780367876555

[B25] Fachner J. Gold C. Erkkilä J. (2013). Music therapy modulates fronto-temporal activity in Rest-EEG in depressed clients. *Brain Topogr.* 26 338–354. 10.1007/s10548-012-0254-x 22983820

[B26] Fachner J. Maidhof C. Grocke D. Nygaard Pedersen I. Trondalen G. Tucek G. (2019). Telling me not to worry…” Hyperscanning and neural dynamics of emotion processing during guided imagery and music. *Front. Psychol.* 10:1561. 10.3389/fpsyg.2019.01561 31402880 PMC6673756

[B27] Fachner J. Maidhof C. Vogl J. Heine A. Steinhoff N. Tucek G. (2021). From the lab to the field«: EEG hyperscanning and qualitative analysis of moments of interest in music therapy for stroke rehabilitation – a study protocol. *Musikther. Umsch.* 42 360–375. 10.13109/muum.2021.42.4.360 PMC9139517 35624953

[B28] Fancourt D. Perkins R. Ascenso S. Carvalho L. A. Steptoe A. Williamon A. (2016). Effects of group drumming interventions on anxiety, depression, social resilience and inflammatory immune response among mental health service users. *PLoS One* 11:e0151136. 10.1371/journal.pone.0151136 26974430 PMC4790847

[B29] Faulkner S. (2017). Rhythm2Recovery: A model of practice combining rhythmic music with cognitive reflection for social and emotional health within trauma recovery. *Aust. N. Z. J. Fam. Ther.* 38 627–636. 10.1002/anzf.1268

[B30] Fitch W. T. (2006). The biology and evolution of music: A comparative perspective. *Cognition* 100 173–215. 10.1016/j.cognition.2005.11.009 16412411

[B31] Foubert K. Collins T. De Backer J. (2017). Impaired maintenance of interpersonal synchronization in musical improvisations of patients with borderline personality disorder. *Front. Psychol.* 8:537. 10.3389/fpsyg.2017.00537 28496420 PMC5407194

[B32] Gugnowska K. Novembre G. Kohler N. Villringer A. Keller P. E. Sammler D. (2022). Endogenous sources of interbrain synchrony in duetting pianists. *Cereb. Cortex* 32 4110–4127. 10.1093/cercor/bhab469 35029645 PMC9476614

[B33] Hadar T. Amir D. (2021). Intimacy, mutuality & negotiations: Dialogic moments in joint improvisation. *Nord. J. Music Ther.* 30 1–25. 10.1080/08098131.2021.1915855

[B34] Hibben J. (1999). *Inside music therapy: Client experiences.* Dallas, TX: Barcelona Publishers.

[B35] Hoehl S. Fairhurst M. Schirmer A. (2020). Interactional synchrony: Signals, mechanisms, and benefits. *Soc. Cogn. Affect. Neurosci.* 16 5–18. 10.1093/scan/nsaa024 32128587 PMC7812629

[B36] Hsu M. H. Flowerdew R. Parker M. Fachner J. Odell-Miller H. (2015). Individual music therapy for managing neuropsychiatric symptoms for people with dementia and their carers: A cluster randomised controlled feasibility study. *BMC Geriatr.* 15:84. 10.1186/s12877-015-0082-4 26183582 PMC4506459

[B37] Kang K. Orlandi S. Lorenzen N. Chau T. Thaut M. H. (2022). Does music induce interbrain synchronization between a non-speaking youth with cerebral palsy (CP), a parent, and a neurologic music therapist? A brief report. *Dev. Neurorehabil.* 25 426–432. 10.1080/17518423.2022.2051628 35341463

[B38] Kelleher R. J. Shen J. (2017). Presenilin-1 mutations and Alzheimer’s disease. *Proc. Natl. Acad. Sci. U.S.A.* 114 629–631. 10.1073/pnas.1619574114 28082723 PMC5278466

[B39] Khalil A. Musacchia G. Iversen J. R. (2022). It takes two: Interpersonal neural synchrony is increased after musical interaction. *Brain Sci.* 12:409. 10.3390/brainsci12030409 35326366 PMC8946180

[B40] King B. (1961). *Stand by me.* New York, NY: Atco.

[B41] Kings of Leon (2008). *Use somebody.* New York, NY: RCA Sony.

[B42] Koelsch S. (2013). From social contact to social cohesion–The 7 Cs. *Music Med.* 5 204–209. 10.1177/1943862113508588

[B43] Kokal I. Engel A. Kirschner S. Keysers C. (2011). Synchronized drumming enhances activity in the caudate and facilitates prosocial commitment - if the rhythm comes easily. *PLoS One* 6:e27272. 10.1371/journal.pone.0027272 22110623 PMC3217964

[B44] Kurkjian A. Skinner K. Ahonen H. (2021). Using music-adapted technology to explore Bruscia’s clinical techniques introduced in autism research: Pilot study. *Approaches* 13 178–204.

[B45] Lindenberger U. Li S.-C. Gruber W. Müller V. (2009). Brains swinging in concert: Cortical phase synchronization while playing guitar. *BMC Neurosci.* 10:22. 10.1186/1471-2202-10-22 19292892 PMC2662862

[B46] Liu L. (2005). *The Chinese neolithic: Trajectories to early states.* Cambridge: Cambridge University Press.

[B47] Luck G. Riikkilä K. Lartillot O. Erkkilä J. Toiviainen P. Mäkelä A. (2006). Exploring relationships between level of mental retardation and features of music therapy improvisations: A computational approach. *Nord. J. Music Ther.* 15 30–48. 10.1080/08098130609478149

[B48] Luck G. Toiviainen P. Erkkilä J. Lartillot O. Riikkilä K. Mäkelä A. (2008). Modelling the relationships between emotional responses to, and musical content of, music therapy improvisations. *Psychol. Music* 36 25–45. 10.1177/0305735607079714

[B49] MacDonald R. A. Wilson G. B. (2014). Musical improvisation and health: A review. *Psychol. WellBeing* 4:20. 10.1186/s13612-014-0020-9

[B50] Malloch S. Trevarthen C. (2009). *Communicative musicality: Exploring the basis of human companionship.* Oxford: Oxford University Press.

[B51] Matusz P. J. Dikker S. Huth A. G. Perrodin C. (2019). Are we ready for real-world neuroscience? *J. Cogn. Neurosci.* 31 327–338. 10.1162/jocn_e_01276 29916793 PMC7116058

[B52] Mayer-Benarous H. Benarous X. Vonthron F. Cohen D. (2021). Music therapy for children with autistic spectrum disorder and/or other neurodevelopmental disorders: A systematic review. *Front. Psychiatry* 12:643234. 10.3389/fpsyt.2021.643234 33897497 PMC8062803

[B53] Mogan R. Fischer R. Bulbulia J. A. (2017). To be in synchrony or not? A meta-analysis of synchrony’s effects on behavior, perception, cognition and affect. *J. Exp. Soc. Psychol.* 72 13–20. 10.1016/j.jesp.2017.03.009

[B54] Müller V. (2022). Neural synchrony and network dynamics in social interaction: A hyper-brain cell assembly hypothesis. *Front. Hum. Neurosci.* 16:848026. 10.3389/fnhum.2022.848026 35572007 PMC9101304

[B55] Müller V. Lindenberger U. (2011). Cardiac and respiratory patterns synchronize between persons during choir singing. *PLoS One* 6:e24893. 10.1371/journal.pone.0024893 21957466 PMC3177845

[B56] Müller V. Lindenberger U. (2019). Dynamic orchestration of brains and instruments during free guitar improvisation. *Front. Integr. Neurosci.* 13:50. 10.3389/fnint.2019.00050 31551723 PMC6738335

[B57] Müller V. Lindenberger U. (2022). Probing associations between interbrain synchronization and interpersonal action coordination during guitar playing. *Ann. N. Y. Acad. Sci.* 1507 146–161. 10.1111/nyas.14689 34510474

[B58] Müller V. Ohström K.-R. P. Lindenberger U. (2021). Interactive brains, social minds: Neural and physiological mechanisms of interpersonal action coordination. *Neurosci. Biobehav. Rev.* 128 661–677. 10.1016/j.neubiorev.2021.07.017 34273378

[B59] Müller V. Sänger J. Lindenberger U. (2013). Intra- and inter-brain synchronization during musical improvisation on the guitar. *PLoS One* 8:e73852. 10.1371/journal.pone.0073852 24040094 PMC3769391

[B60] Müller V. Sänger J. Lindenberger U. (2018). Hyperbrain network properties of guitarists playing in quartet: Hyperbrain networks during quartet playing. *Ann. N. Y. Acad. Sci.* 1423 198–210. 10.1111/nyas.13656 29543978

[B61] Noguchi K. Gel Y. R. Brunner E. Konietschke F. (2012). **nparLD**: An *R* software package for the nonparametric analysis of longitudinal data in factorial experiments. *J. Stat. Softw.* 50 1–23. 10.18637/jss.v050.i1225317082

[B62] Nordoff P. Robbins C. (1977). *Creative music therapy: Individualized treatment for the handicapped child.* New York, NY: John Day Company.

[B63] Nozaradan S. (2014). Exploring how musical rhythm entrains brain activity with electroencephalogram frequency-tagging. *Philos. Trans. R. Soc. B Biol. Sci.* 369:20130393. 10.1098/rstb.2013.0393 25385771 PMC4240960

[B64] Nozaradan S. Zerouali Y. Peretz I. Mouraux A. (2015). Capturing with EEG the neural entrainment and coupling underlying sensorimotor synchronization to the beat. *Cereb. Cortex* 25 736–747. 10.1093/cercor/bht261 24108804

[B65] Our Dementia Choir (2019). *Our dementia choir with Vicky McClure.* Available online at: https://www.bbc.co.uk/programmes/m0004pyd (accessed September 12, 2022).

[B66] R Core Team (2021). *R: A language and environment for statistical computing.* Available online at: https://www.R-project.org/ (accessed October 11, 2022).

[B67] Reznik S. J. Allen J. J. B. (2018). Frontal asymmetry as a mediator and moderator of emotion: An updated review. *Psychophysiology* 55:e12965. 10.1111/psyp.12965 28776710

[B68] Rojiani R. Zhang X. Noah A. Hirsch J. (2018). Communication of emotion via drumming: Dual-brain imaging with functional near-infrared spectroscopy. *Soc. Cogn. Affect. Neurosci.* 13 1047–1057. 10.1093/scan/nsy076 30215809 PMC6204489

[B69] Samadani A. Kim S. Moon J. Kang K. Chau T. (2021). Neurophysiological synchrony between children with severe physical disabilities and their parents during music therapy. *Front. Neurosci.* 15:531915. 10.3389/fnins.2021.531915 33994913 PMC8119766

[B70] Sänger J. Lindenberger U. Müller V. (2011). Interactive brains, social minds. *Commun. Integr. Biol.* 4 655–663. 10.4161/cib.17934 22448303 PMC3306325

[B71] Sänger J. Müller V. Lindenberger U. (2012). Intra- and interbrain synchronization and network properties when playing guitar in duets. *Front. Hum. Neurosci.* 6:312. 10.3389/fnhum.2012.00312 23226120 PMC3509332

[B72] Sänger J. Müller V. Lindenberger U. (2013). Directionality in hyperbrain networks discriminates between leaders and followers in guitar duets. *Front. Hum. Neurosci.* 7:234. 10.3389/fnhum.2013.00234 23761745 PMC3671173

[B73] Schilbach L. (2016). Towards a second-person neuropsychiatry. *Philos. Trans. R. Soc. B Biol. Sci.* 371:20150081. 10.1098/rstb.2015.0081 26644599 PMC4685526

[B74] Schirmer A. Fairhurst M. Hoehl S. (2021). Being ‘in sync’—is interactional synchrony the key to understanding the social brain? *Soc. Cogn. Affect. Neurosci.* 16 1–4. 10.1093/scan/nsaa148 33104804 PMC7812616

[B75] Sittler M. C. Worschech F. Wilz G. Fellgiebel A. Wuttke-Linnemann A. (2021). Psychobiological mechanisms underlying the health-beneficial effects of music in people living with dementia: A systematic review of the literature. *Physiol. Behav.* 233:113338. 10.1016/j.physbeh.2021.113338 33497696

[B76] Smetana M. Stepniczka I. Bishop L. (2022). COME_IN: A qualitative framework for content, meanings and intersubjectivity in free dyadic improvisations. *Nord. J. Music Ther.* 32 157–178. 10.1080/08098131.2022.2084638

[B77] Smith E. E. Reznik S. J. Stewart J. L. Allen J. J. B. (2017). Assessing and conceptualizing frontal EEG asymmetry: An updated primer on recording, processing, analyzing, and interpreting frontal alpha asymmetry. *Int. J. Psychophysiol.* 111 98–114. 10.1016/j.ijpsycho.2016.11.005 27865882 PMC6449497

[B78] Sun L. Zhou R. Yang G. Shi Y. (2017). Analysis of 138 pathogenic mutations in presenilin-1 on the in vitro production of Aβ42 and Aβ40 peptides by γ-secretase. *Proc. Natl. Acad. Sci. U.S.A.* 114 E476–E485. 10.1073/pnas.1618657114 27930341 PMC5278480

[B79] Toiviainen P. (2001). Real-time recognition of improvisations with adaptive oscillators and a recursive bayesian classifier. *J. New Music Res.* 30 137–147. 10.1076/jnmr.30.2.137.7112

[B80] Toiviainen P. Snyder J. S. (2003). Tapping to bach: Resonance-based modeling of pulse. *Music Percept.* 21 43–80. 10.1525/mp.2003.21.1.43

[B81] Trost W. Labbé C. Grandjean D. (2017). Rhythmic entrainment as a musical affect induction mechanism. *Neuropsychologia* 96 96–110. 10.1016/j.neuropsychologia.2017.01.004 28069444

[B82] Tucek G. Maidhof C. Vogl J. Heine A. Zeppelzauer M. Steinhoff N. (2022). EEG hyperscanning and qualitative analysis of moments of interest in music therapy for stroke rehabilitation—A feasibility study. *Brain Sci.* 12:24. 10.3390/brainsci12050565 35624953 PMC9139517

[B83] Van Noorden L. Moelants D. (1999). Resonance in the perception of musical pulse. *J. New Music Res.* 28 43–66.

[B84] Wigram T. (2004). *Improvisation: Methods and techniques for music therapy clinicians, educators, and students.* London: Jessica Kingsley Publishers.

[B85] Will U. Makeig S. (2011). “EEG research methodology and brainwave entrainment,” in *Music, science and the rhythmic brain. Cultural and clinical implications*, eds Turow G. Berger J. (New York, NY: Routledge), 86–107.

[B86] Winkelman M. (2003). Complementary therapy for addiction: “drumming out drugs.” *Am. J. Public Health* 93 647–651. 10.2105/AJPH.93.4.647 12660212 PMC1447805

[B87] Wittenburg P. Brugman H. Russel A. Klassmann A. Sloetjes H. (2006). “ELAN: A professional framework for multimodality research,” in *Proceedings of the fifth international conference on language resources and evaluation (LREC’06)*, Vol. 4 Genoa.

[B88] Wosch T. Wigram T. eds (2007). *Microanalysis in music therapy: Methods, techniques and applications for clinicians, researchers, educators and students.* London: Jessica Kingsley Publishers.

[B89] Zamm A. Debener S. Bauer A.-K. R. Bleichner M. G. Demos A. P. Palmer C. (2018). Amplitude envelope correlations measure synchronous cortical oscillations in performing musicians: Amplitude envelopes measure inter-brain synchrony. *Ann. N. Y. Acad. Sci.* 1423 251–263. 10.1111/nyas.13738 29756657

[B90] Zoefel B. ten Oever S. Sack A. T. (2018). The involvement of endogenous neural oscillations in the processing of rhythmic input: More than a regular repetition of evoked neural responses. *Front. Neurosci.* 12:95. 10.3389/fnins.2018.00095 29563860 PMC5845906

